# Pharmacokinetic-pharmacodynamic analysis of drug liking blockade by buprenorphine subcutaneous depot (CAM2038) in participants with opioid use disorder

**DOI:** 10.1038/s41386-023-01793-z

**Published:** 2024-01-10

**Authors:** Sharon L. Walsh, Sandra D. Comer, Jurij Aguiar Zdovc, Céline Sarr, Marcus Björnsson, Kerstin Strandgården, Peter Hjelmström, Fredrik Tiberg

**Affiliations:** 1 Behavioral Science, Pharmacology, Psychiatry and Pharmaceutical Sciences Departments, University of Kentucky College of Medicine and Pharmacy, Kentucky, KY USA; 2 Department of Psychiatry, Columbia University, New York, NY USA; 3 Pharmetheus AB, Uppsala, Sweden; 4 Camurus AB, Lund, Sweden; 5 Uppsala Monitoring Centre, Uppsala, Sweden

**Keywords:** Addiction, Translational research, Drug development

## Abstract

Buprenorphine is used to treat opioid use disorder (OUD). Weekly and monthly subcutaneous long-acting buprenorphine injections (CAM2038) provide more stable buprenorphine plasma levels and reduce the treatment burden, misuse, and diversion associated with sublingual transmucosal buprenorphine formulations. To characterize the pharmacokinetic/pharmacodynamic (PK/PD) relationship, a maximum inhibition (I_max_) model was developed relating CAM2038 buprenorphine plasma concentration to drug liking maximum effect (E_max_) visual analog scale (VAS; bipolar) score after intramuscular hydromorphone administration. Data included time-matched observations of buprenorphine plasma concentration and drug liking E_max_ VAS score after hydromorphone 18 mg administration in 47 non-treatment-seeking adults with moderate to severe OUD in a phase 2 study. Analysis used non-‍linear mixed-effects modeling (NONMEM^®^). The final I_max_ model adequately described the PK/PD relationship between buprenorphine plasma concentration and drug liking E_max_ VAS score. Simulations showed drug liking was effectively blocked at low buprenorphine plasma concentrations (0.4 ng/mL) where the upper 95% confidence interval of the drug liking E_max_ VAS score was below the pre-defined 11-point complete blockade threshold. The buprenorphine plasma concentration required to achieve 90% of the maximal effect (IC_90_) of drug liking was 0.675 ng/mL. Interindividual variability in responses to buprenorphine was observed; some participants experienced fluctuating responses, and a few did not achieve drug liking blockade even with higher buprenorphine plasma concentrations. This affirms the need to individualize treatment and titrate doses for optimal treatment outcomes. PK/PD models were also developed for desire to use VAS and Clinical Opiate Withdrawal Scale (COWS) scores, with results aligned to those for drug liking.

## Introduction

Illicit opioid use continues to rise, with approximately 61 million people globally using opioids for non-medical purposes in 2020 [1]. Over 16 million individuals suffer from opioid use disorder (OUD), whereby opioids are chronically used despite significant distress or impairment, resulting in substantial clinical, economic, and societal burden [1–3].

Buprenorphine (BPN) is an efficacious and widely used opioid to treat pain and OUD [4–6]. In OUD, BPN suppresses opioid withdrawal and cravings, reduces illicit opioid use, and blocks the effects of exogenous opioids through its actions as a partial μ-opioid receptor agonist with high-affinity and slow dissociation binding to μ-opioid receptors [5, 7]. As a result of the partial μ-opioid receptor agonism, there is a ceiling effect for μ-opioid receptor-mediated effects such as respiratory depression and euphoria, resulting in a better safety profile and reduced potential for misuse compared to full μ-opioid receptor agonists like methadone [4].

BPN is typically administered as daily transmucosal sublingual tablets or films and has been shown to reduce illicit opioid use, retain patients in treatment, and reduce mortality [4, 8]. Two long-acting injections of BPN (LAIBs) have been recently introduced. LAIBs aim to provide more stable BPN plasma levels, extend dosing intervals, improve treatment adherence, and reduce the misuse and diversion associated with sublingual BPN formulations [9, 10]. CAM2038 is a subcutaneous LAIB available in weekly (Q1W) and monthly (Q4W) doses [4, 11–14]. CAM2038 provides more steady BPN release than standard daily sublingual BPN treatment [4, 13]. Randomized clinical trials of CAM2038 have also demonstrated superior reduction of overall opioid use, improved treatment satisfaction and quality of life, and reduced treatment burden versus daily sublingual therapy [9, 10].

Complete (100%) absolute bioavailability of BPN is achieved with subcutaneous CAM2038 formulations compared with the much lower bioavailability after sublingual administration (21–29%) [13]; this is mainly due to the first-pass metabolism of the swallowed fraction of BPN. Initial BPN release from the CAM2038 formulations is relatively rapid, with median time to maximum BPN plasma concentration of approximately 24 h and 4–10 h for CAM2038 Q1W and Q4W, respectively [4, 13]. Due to the continuous and gradual release of BPN from the CAM2038 depot matrix and absorption into systemic circulation, the plasma half-life of BPN is longer following subcutaneous administration (CAM2038 Q1W: approximately 5 days; Q4W: 19–25 days) than after sublingual administration (24–42 h) [5, 13]. Therefore, CAM2038 provides more stable BPN release than the daily sublingual treatment, which eliminates the peaks and troughs associated with daily administration [4, 5, 13].

As subcutaneous BPN bypasses first-pass metabolism associated with sublingual administration, CAM2038 also benefits from reduced and more stable norbuprenorphine (a full μ-opioid receptor agonist) levels compared to sublingual BPN [5, 15]. Norbuprenorphine, a BPN metabolite, may be associated with a greater risk of respiratory depression than BPN [5, 13, 15].

One of the aforementioned clinical trials that assessed the efficacy of CAM2038 24 mg and 32 mg Q1W among participants with OUD was a phase 2 study that demonstrated an immediate and sustained blockade of opioid agonist effect and withdrawal suppression with BPN [4]. CAM2038 also produced dose-proportional BPN plasma concentration-time profiles with increased BPN plasma concentrations following successive injections [4]. Some studies have suggested that a BPN plasma concentration of 2–3 ng/mL is required for significant blockade of opioid drug liking [7, 16–18]. However, this phase 2 study of CAM2038 reported blockade of opioid agonist effect with BPN plasma concentrations of approximately 1.25 ng/mL [4]. This result agrees with previous studies that indicated a BPN plasma concentration as low as 0.7 ng/mL, or a μ-opioid receptor occupancy of at least 50–60%, is required for suppression of opioid withdrawal and agonist symptoms, while suppression of craving might occur at lower BPN plasma concentrations [19–21].

This model analysis aimed to further characterize the PK/PD relationship between BPN plasma concentration and drug liking with CAM2038 Q1W using data from the phase 2 study. PK/PD analyses for two secondary endpoints, measuring desire to use and opioid withdrawal suppression, were also performed. Because relative norbuprenorphine to BPN exposure at steady state after CAM2038 administration is 5- to 7-‍fold lower than after sublingual BPN administration (due to first-pass metabolism of BPN to norbuprenorphine with sublingual administration; unpublished data), the potential role of norbuprenorphine was not assessed here.

## Materials and methods

### Data

This PK/PD analysis utilized data collected in a phase 2, multisite, randomized, double-blind, repeat-dose study (NCT02611752) that evaluated the ability of CAM2038 to block opioid drug liking effects and suppress opioid withdrawal when non-treatment-seeking, healthy adults with moderate to severe OUD (*N* = 47) were challenged with hydromorphone. The study design has been reported previously (Supplementary Fig. S1) [4]. Following screening and qualification, participants (mean [standard deviation] age, 35.8 [9.1] years; 35 [74%] male; 24 [51%] Black or African American; further baseline characteristics provided in Table S1) were randomized (1:1), stratified by sex, to receive subcutaneous CAM2038 24 mg or 32 mg Q1W (days 0 and 7). During qualification (days –3 to –1) and on days 1–3, 4–6, 8–10, and 11–13, hydromorphone was administered intramuscularly once daily over each 3-day period (0, 6, or 18 mg; order randomized across participants but consistent across all 3-day periods for each participant; only data from the 18 mg challenge sessions were included here).

Plasma samples to quantify BPN concentration were collected approximately 60 min before each hydromorphone administration. Drug liking maximum effect (E_max_) visual analog scale (VAS) scores were collected 30 min before hydromorphone administration (challenge session) and at 5, 10, 15, 30, 45, 60, 75, 90, 120, 150, 180, 210, 240, 270, and 300 min post-hydromorphone administration. A bipolar VAS score was used, with scores ranging from 0–100 (50 indicated a neutral response) [4], as requested by the US Food and Drug Administration (FDA) pre-study. Although some aspects of a unipolar scale may be preferable to a bipolar scale [22], this was not used due to the FDA’s request.

The trial protocol was reviewed and approved by site ethics committees and conducted in accordance with the Declaration of Helsinki and International Council for Harmonization (ICH) Good Clinical Practice (GCP) and any applicable national and local laws and regulations. The Institutional Review Board (IRB) or Independent Ethics Committee (IEC) included Midlands Independent Review Board (A WIRB-Copernicus Group Company), U of Kentucky IRB #1, and New York State Psychiatric Institute IRB. All participants provided written informed consent.

Details on data collection, modeling, and analysis for the desire to use VAS score and Clinical Opiate Withdrawal Scale (COWS) score are presented in the Supplementary Methods; methods hereafter refer to the primary PK/PD analysis for drug liking.

Available PK and PD data for the analysis included the daily measure (one sample measure per challenge) of BPN plasma concentration collected 60 min before hydromorphone challenge and drug liking E_max_ VAS scores. Drug liking E_max_ VAS scores were period-corrected whereby the post-hydromorphone challenge score was subtracted from the pre-challenge value. The analysis dataset included 231 data points (231 time-matched measurements of BPN plasma concentrations and drug liking E_max_ VAS scores) from 47 participants collected during hydromorphone 18 mg challenge sessions (analysis datasets provided in Table S2). Data from the hydromorphone 0 mg and 6 mg challenges were excluded during the PK/PD modeling because these participants had no (0 mg) or lower (6 mg) drug liking responses compared to after hydromorphone 18 mg challenge. Therefore, 18 mg challenge provided the most informative results.

### Model development

A direct maximum inhibition (I_max_) model relating BPN plasma concentration to period-corrected drug liking E_max_ VAS score was developed using these data. A structural model, describing the main trends, and models for interindividual variability (IIV) and residual unexplained variability (RUV), were included. Parameter estimation was performed using the first-order conditional estimation with interaction (FOCEI) method in non-linear mixed-effects modeling (NONMEM^®^) and the standard errors of the parameter estimates were computed using the default MATRIX option in the NONMEM^®^ $COV record.

The starting point for the structural I_max_ model is described below; VAS(Cp) is the period-corrected drug liking E_max_ VAS function of the daily BPN plasma concentration at the time of the efficacy measurement, BASE is the period-corrected drug liking E_max_ VAS score before CAM2038 administration, I_max_ is the maximum inhibitory effect of BPN, IC_50_ is the BPN plasma concentration required to achieve half of the maximum effect, and γ is the sigmoidicity parameter.

$${{{{{\rm{VAS}}}}}}({{{{{\rm{Cp}}}}}})={{{{{\rm{BASE}}}}}}\cdot \left(1-\frac{{{{{{{\rm{I}}}}}}}_{\max }\cdot {{{{{\rm{C}}}}}}{{{{{{\rm{p}}}}}}}^{{{{{{\rm{\gamma }}}}}}}}{{{{{{{\rm{IC}}}}}}}_{50}^{{{{{{\rm{\gamma }}}}}}}+{{{{{\rm{C}}}}}}{{{{{{\rm{p}}}}}}}^{{{{{{\rm{\gamma }}}}}}}}\right)$$

The BPN plasma concentration required to achieve 90% of the maximal effect (IC_90_) was also estimated as it is a more relevant clinical target compared to the common pharmacology endpoint of IC_50_.

Log-normal distributions were initially evaluated for each parameter, with other distributions including additive, logit, and Box-Cox transformations also tested (Supplementary Methods). The final analysis used a logit-transformation with an additive RUV model enforcing predictions to fit between –1 and 52 (Supplementary Methods).

### Covariate model building

The influence of sex, age, race, ethnicity, body weight, height, and body mass index (BMI) were tested on IC_50_ of drug liking, one at a time, by a single forward step using the stepwise covariate model building procedure in Perl-speaks-NONMEM^®^ [23, 24]. Continuous covariate relationships were coded as power models and categorical covariates were generally coded as a fractional difference to the most common category. The effect of baseline drug liking E_max_ VAS score on IC_50_ was explored using an omega block in NONMEM^®^. Covariate relationships were also explored graphically by plotting IIV versus covariates.

### Simulations

Exposure-response relationship simulations, including parameter uncertainty, were performed by sampling 10,000 sets of parameters using the covariance matrix. The typical value of drug liking E_max_ VAS score was calculated alongside the median and 95% confidence interval (CI) for each parameter set and simulated concentration.

### Model evaluation

Model evaluation was based on the inspection of graphical diagnostics, including goodness of fit plots, visual predictive checks (VPCs), and between-model changes in objective function values provided by NONMEM^®^ (Supplementary Methods).

For VPCs, data were simulated 1000 times using the doses and covariate data from the participants included in the analysis dataset. Graphical comparison of observed and simulated dependent variables versus time or concentration was conducted, and a 95% CI was used. Discrimination between models was mainly based on the inspection of graphical diagnostics and changes in the objective function values provided by NONMEM^®^.

### Software

Analyses were performed using NONMEM^®^ version 7.3.0. VPCs and log-likelihood profiling were run using PsN version 4.4.8 [23, 25]. Xpose version 4.5.3 was used as an aid in model assessment [26]. Additional details are provided in the Supplementary Methods.

## Results

### Observed PK/PD response

**Fig. 1:**
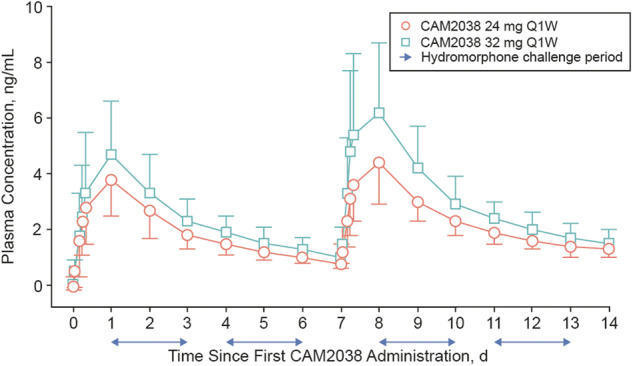
BPN plasma concentration-time profile. Graph shows the arithmetic mean (±1 SD) BPN plasma concentrations for the cohorts over the course of the study. CAM2038, 24 mg: *n* = 22; 32 mg: *n* = 24. BPN buprenorphine, d day, Q1W once weekly, SD standard deviation. Figure adapted from Walsh et al. [4].

A dose-proportional increase in BPN plasma concentration-time profile was achieved with CAM2038 Q1W (24 mg and 32 mg), and higher BPN plasma concentrations were achieved after the second dose of CAM2038 (Fig. 1) [4]. PK profiles were consistent across individuals (Supplementary Fig. S2).

**Fig. 2:**
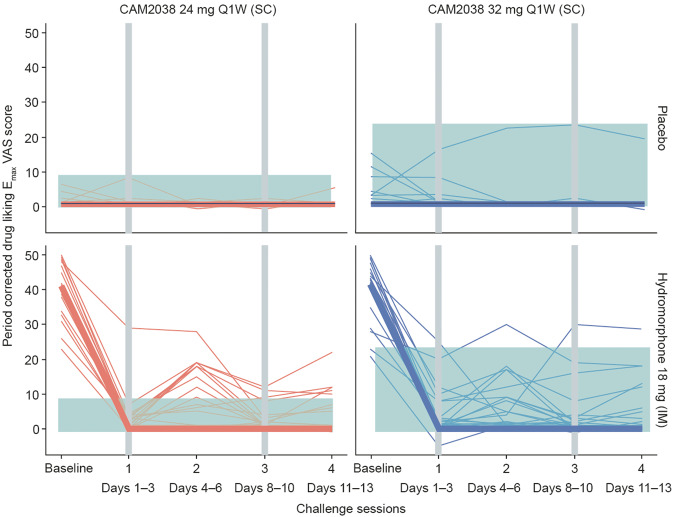
Observed drug liking E_max_ VAS score versus hydromorphone challenge sessions. Observed period-corrected drug liking E_max_ VAS score versus challenge sessions, stratified by the dose at randomization and the hydromorphone challenge dose. Each thin line represents one participant and is colored by dose group. The thick line represents the geometric mean for the panel. The shaded area represents the range of drug liking scores observed in the intramuscular placebo challenge dose condition. The vertical gray lines indicate the CAM2038 administrations. E_max_ maximum effect, IM intramuscular, Q1W once weekly, SC subcutaneous, VAS visual analog scale.

Prior to CAM2038 administration, a strong drug liking E_max_ VAS response to hydromorphone 18 mg challenge was measured. This was effectively suppressed after the first dose of CAM2038, with further suppression following the second dose (Fig. 2). No carry-over effect of the challenges (placebo, hydromorphone 6 mg, hydromorphone 18 mg) was observed from previous periods within each session. Similar, relatively rapid suppressions of desire to use VAS and COWS scores versus BPN plasma concentration and time were also observed (Supplementary Data).

At the individual level, BPN plasma concentrations were higher and consequently, drug liking E_max_ VAS scores following hydromorphone 18 mg challenge were lower after the second dose of CAM2038 compared with the first dose for most participants (Supplementary Fig. S3). No hysteresis in the relationship between BPN plasma concentration and drug liking E_max_ VAS scores was observed.

### Model development

The starting model was an I_max_ model linking daily BPN plasma concentrations to daily period-corrected drug liking E_max_ VAS scores, collected after hydromorphone 18 mg challenge (described in Methods). I_max_ and γ were fixed to 1, but all other parameters were estimated. IIVs were estimated only on BASE and IC_50_, and the RUV was described by a combined error model.

To avoid excessive negative drug liking E_max_ VAS scores in the simulations and an over-prediction of post-CAM2038 administration scores, a logit transformation of the predictions was used so predictions could fit in the range –1 to 52. This boundary allowed for model stability and predictions covering the entire range of observations (few [3/231] observations were <0). Estimation of the combined error model showed that the proportional component of the error model was no longer relevant, so only the additive error model was pursued. As shown in the goodness of fit plots and VPCs, the additive error model could capture the baseline IIV and the post-dose points. The BASE IIV distribution and IC_50_ IIV distribution of this run were not normally distributed but testing different shapes did not improve the fit and provided higher uncertainty on parameter estimates. Consequently, this was selected as the final model. Desire to use VAS and COWS score model development is outlined in the Supplementary Methods.

### Final PK/PD models

**Fig. 3:**
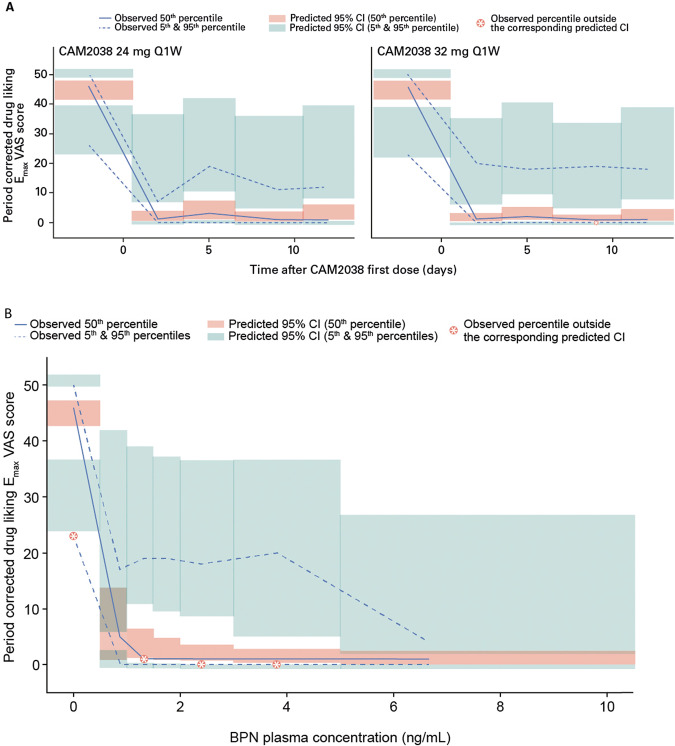
Visual predictive check of drug liking E_max_ VAS score. **A** Visual predictive check of period-corrected drug liking E_max_ VAS score versus time, stratified by dose. **B** Visual predictive check of period-corrected drug liking E_max_ VAS score versus BPN concentration. Drug liking score was period corrected, whereby the pre-challenge value was subtracted from the maximum value recorded after each 18 mg hydromorphone challenge. The solid and dashed blue lines represent the median, 5th and 95th percentiles of the observations; the shaded red and blue areas represent the 95% confidence interval of the median, 5th and 95th percentiles predicted by the model. BPN buprenorphine, CI confidence interval, E_max_ maximum effect, Q1W once weekly, VAS visual analog scale.

The final I_max_ models adequately described the PK/PD relationships between BPN plasma concentration and drug liking E_max_ VAS, desire to use VAS, and COWS scores. Goodness of fit plots (Supplementary Fig. S4) for the final drug liking E_max_ VAS score model and VPCs of each endpoint versus time and versus BPN plasma concentration indicated good model performance (Fig. 3 and Supplementary Figs. S5, S6). The population PK/PD model parameters estimated with the final BPN models are presented in Table 1 and Tables S3, S4. All standard errors for the drug liking E_max_ VAS score model were less than 31% and shrinkage less than 14%. No statistically significant effect of age, sex, ethnicity, race, body weight, height, or BMI was found for IC_50_ (*p* > 0.2). The drug liking IC_90_ estimate was 0.675 ng/mL. The IC_90_ estimates for desire to use VAS and COWS scores were 0.116 and 0.109 ng/mL, respectively.

**Table 1:** Parameter estimates of the final PK/PD models.

	Unit	Value	RSE (%)	Shrinkage (%)
Drug liking E_max_ VAS score				
Baseline	VAS units	45.3	2.40	
IC_50_	ng/mL	0.075	30.4	
I_max_		1.00	(FIX)	
IIV Baseline	SD	0.106	19.8	11.6
IIV IC_50_	SD	1.90	10.3	13.3
IIV I_max_	SD	0	(FIX)	
Additive RUV (logit scale)		0.744	8.62	9.39
	Drug liking E_max_ VAS score	Desire to use VAS score		COWS score
IC_90_ (ng/mL)	0.675	0.116		0.109

The relative standard error (RSE) for interindividual variability (IIV) is reported on the approximate standard deviation (SD) scale. The relative RSE for residual unexplained variability (RUV) is reported on the approximate SD on the normal scale. The parameters without RSE are fixed.

*COWS* Clinical Opiate Withdrawal Scale, *E*_*max*_ maximum effect, *IC*_*50*_ concentration at half maximum inhibition, *IC*_*90*_ concentration at 90% of maximum inhibition, *I*_*max*_ concentration at maximum inhibition, *IIV* interindividual variability, *PD* pharmacodynamic, *PK* pharmacokinetic, *RSE* relative standard error, *RUV* residual unexplained variability, *SD* standard deviation, *VAS* visual analog scale.

**Fig. 4:**
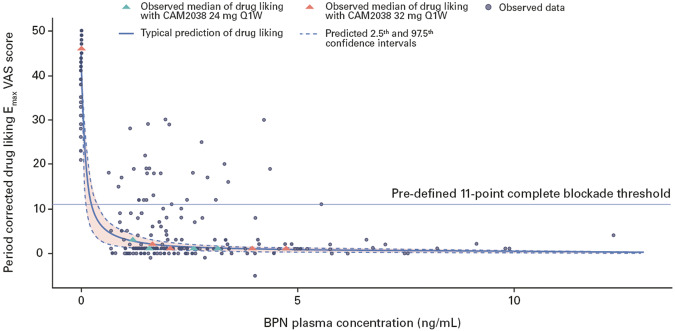
Period-corrected drug liking E_max_ VAS score versus BPN plasma concentration. Observed period-corrected drug liking E_max_ VAS score includes data from hydromorphone 18 mg challenge sessions at randomization (before CAM2038 administration) and data from subsequent 18 mg hydromorphone challenge sessions after CAM2038 administration. The circles represent observed data, and the triangles are the observed median of period-corrected drug liking E_max_ VAS score for the corresponding mean BPN plasma concentrations. Medians of observed data are shown since the drug liking E_max_ VAS scores at a given concentration or concentration range were not normally distributed. The solid and dashed blue lines represent the typical prediction, and 2.5th and 97.5th confidence intervals (shaded) of the model predictions, simulated with parameter uncertainty. The median value was selected for comparison to simulations due to the high number of zero values in the dataset. BPN buprenorphine, E_max_ maximum effect, Q1W once weekly, VAS visual analog scale.

Simulations were performed, based on the final E_max_ VAS score model, to illustrate the PK/PD relationship (Fig. 4). The model predicted the phase 2 study results well. Based on the upper 95% CI of the simulated drug liking E_max_ VAS score intercepting the pre-defined 11-point complete blockade threshold (Fig. 4) [27], drug liking was effectively blocked at a BPN plasma concentration of 0.4 ng/mL (differing from the IC_90_ parameter estimate of 0.675 ng/mL). Consistent with the PK/PD model, medians of observed data showed complete blockade of drug liking E_max_ VAS score at relatively low BPN plasma concentrations.

There was some IIV in drug liking E_max_ VAS scores, and higher BPN plasma concentration did not result in complete blockade for some participants (Fig. 4). Some participants demonstrated greater drug liking blockade with increased BPN plasma concentration but did not reach the 11-point complete blockade threshold. A few participants showed fluctuations in their response to treatment, where increasing BPN plasma concentration did not necessarily increase drug liking blockade effect. For one participant, all recorded drug liking E_max_ VAS scores were above the complete blockade threshold whereby no sufficient response occurred, even at higher BPN plasma concentrations.

The PK/PD relationship between BPN plasma concentration and COWS score was also simulated (Supplementary Fig. S7). The predicted drug efficacy was high; at the BPN plasma concentration of 0.100 ng/mL, 99.5% of the simulated patients had a COWS score <5 and 80.4% of the simulated patients had a COWS score ≤1. This is aligned with the IC_90_ estimate of 0.109 ng/mL, less than the lowest observed BPN plasma concentration of 0.636 ng/mL.

## Discussion

The final I_max_ models adequately described the exposure-response relationship between BPN plasma concentration and drug liking E_max_ VAS, desire to use VAS, and COWS scores for CAM2038. The estimated IC_90_ for the drug liking E_max_ VAS score was relatively low (0.675 ng/mL) while simulated typical values indicated that drug liking was effectively blocked at a lower BPN plasma concentration (0.4 ng/mL). These values are lower than previously reported concentrations required for blockade of opioid effects with subcutaneous and sublingual BPN formulations [7, 16–18]. Additionally, simulations based on participant data predicted nearly all patients to have a COWS score lower than 5, indicating mild symptoms, at a BPN plasma concentration of 0.1 ng/mL.

Although clinical dosing decisions are not typically reliant on plasma concentrations, these estimates may increase clinicians’ awareness of target BPN plasma concentrations during OUD treatment and the expected IIV. The observed IIV in the relationship between BPN plasma concentration and achievement of drug liking blockade affirms the need to titrate doses, and the requirement for individualized treatment depending on participants’ response (for example, via alternative formulations and dose strengths). As this model suggests, individualized treatment could provide benefit for patients who experience greater than expected response at elevated BPN plasma concentrations; however, it would not necessarily improve the response for those experiencing variable efficacy with increasing BPN plasma concentration. A small number of patients may not experience drug liking blockade despite higher BPN plasma concentrations or individualized treatment for as yet unknown reasons.

Drug liking blockade was used as a clinically meaningful primary outcome as it reflects the ability of a therapeutic to attenuate or fully block the effects of opioids, thus reducing illicit use. This effect can be achieved through blockade with an opioid agonist or partial agonist (i.e., methadone or BPN), or through true pharmacological antagonism (i.e., with naltrexone) [8]. However, numerous patient-level and structural factors, beyond pharmacological blockade, contribute to the overall response to OUD treatment [8]. Nevertheless, this study predicts BPN plasma concentrations above 0.4 ng/mL (simulated complete blockade threshold) or 0.675 ng/mL (IC_90_) may confer desired clinical efficacy to effectively reduce drug liking, aligning with previous analyses of outpatient clinical studies and prescription claims data that found a dose-response in reduction of illicit opioid use [28, 29]. Together, these findings indicate that a dose of at least 8 mg sublingual BPN may be needed to avoid relapse to drug use.

Compared with the drug liking E_max_ VAS score IC_90_ of 0.675 ng/mL, the IC_90_ estimates for desire to use VAS and COWS scores were lower (0.116 and 0.109 ng/mL, respectively) and below the lowest observed BPN plasma concentration in the phase 2 study (0.636 ng/mL). These data align with previous findings that higher BPN concentrations are required for drug liking blockade than for suppression of opioid cravings and withdrawal [16]. Thus, treatment should be individualized based on clinician assessment of not only craving and withdrawal, but also whether the patient is continuing to use opioids and liking their effects despite BPN treatment. In such cases, an increased dose of BPN may be needed.

The lack of observations at very low BPN plasma concentrations limited the precision and certainty of the IC_90_ parameter estimates. No covariate relationships were observed for the drug liking E_max_ VAS score IC_50_. The shrinkage associated with IIV allowed interpretation of the plots of IIV versus covariates with confidence and no obvious trends were detected. However, the population was relatively small (*N* = 47) and a larger population would be required to exclude any covariate relationships.

The ceiling effect observed with higher BPN plasma concentrations in this model is similar to findings from studies of sublingual BPN formulations, where no additional opioid blockade effect has been reported for daily sublingual BPN doses ≥16 mg [7]. Indeed, the majority of studies including tablet doses of BPN 8 and 16 mg reported equivalent effectiveness at such doses [7]. BPN trough plasma concentrations (C_trough_) (geometric mean [CV%]) for these sublingual doses have been reported in a separate study at 0.26 (35.2) and 0.37 (43.8) ng/mL for 8 and 16 mg, respectively, after the first dose, increasing to 0.68 (52.0) and 1.05 (45.6) ng/mL for 8 and 16 mg, respectively, after the seventh dose (i.e., at steady state) [13]. This agrees well with the blocking threshold concentrations obtained by modeling and simulations in this study. However, recent pharmacological and modeling studies have shown there may be situations where use of higher BPN doses can be beneficial; for instance, during induction to quickly maximize BPN agonist effects and avoid precipitating withdrawal in patients using full µ-opioid agonists [30].

Higher BPN plasma concentrations might be needed for blockade of higher opioid doses or opioids with greater potency, such as fentanyl [31, 32]. However, larger outcome studies are needed to confirm whether a dose-dependent relationship for BPN in reducing overdose-related mortality exists, considering that higher doses of BPN might also be associated with opioid-related adverse drug reactions, like central sleep apnea [33]. A recent, large case-crossover study using prescription claims data did not find a difference between high- or low-dose BPN and the risk of non-fatal overdoses with BPN and benzodiazepine combinations [34]. Thus, the dose in OUD treatment should be individualized based on continuous benefit-risk assessment over time [8].

The drug liking E_max_ VAS score PK/PD model showed drug liking inhibition after initial injection, and the combined rapid and sustained release of BPN offers the potential for initiation and long-term maintenance treatment with CAM2038 formulations. CAM2038 offers a more stable BPN plasma concentration over time compared with daily sublingual formulations [13], and this analysis showed a gradual decrease in BPN plasma concentration and suppression of drug liking over a week after the first dose of CAM2038 Q1W. The model captured this behavior over time using only BPN plasma concentration as a driver of efficacy, as shown by the absence of trends in residuals. These findings suggest CAM2038 may provide a more consistent treatment effect over time and could improve adherence versus daily medications [13]. This effect would be reinforced with repeated dosing due to accumulation of BPN plasma concentrations.

The LAIB CAM2038 formulation, therefore, inhibits drug liking at low BPN plasma concentrations and provides a more stable effect over time. The different dosing options of CAM2038 could provide patients with the flexibility to individualize treatment depending on their stage of BPN maintenance treatment [13]. The results of this PK/PD analysis could also help guide clinicians to prescribe an optimal dosing regimen to achieve and maintain therapeutic effects in individual patients.

### Limitations

As with previous opioid blockade studies, this study was based on a small sample size. However, this study did not examine effects of salient/common drug-use history variables (e.g., injection opioid use, cocaine use, alcohol use, smoking history), which, in previous opioid blockade studies influenced variability in outcomes [16].

While this study included high BPN plasma concentrations, analysis of lower doses would be needed to achieve more precise IC_90_ estimates. Additionally, non-normal distributions of modeling parameters were found, which could potentially influence the standard errors of the parameters, however other tested distributions did not fit the data better. A large inter-individual variability was observed in the IC_50_ values for desire to use VAS score, leading to lower precision in the model. This variability makes it difficult to make any predictions of desire to use VAS score on an individual level.

The relationship between the 11-point complete blockade threshold and the actual clinical response and reduction in opioid use in a real-world setting is unknown, despite being the standard threshold accepted by the US FDA [27]. For example, some patients could satisfy the 11-point threshold but may continue opioid use. Additionally, our phase 2 data were collected under controlled conditions not representative of the real world. Therefore, caution should be taken when interpreting and applying these results in clinical practice. The finding of a low threshold BPN plasma concentration, <1 ng/mL, was based on modeling of the data used in this study, while higher concentration thresholds have been reported in other studies [7, 16–18]. It will be important to replicate these findings and to extend them to populations who are largely using fentanyl and fentanyl analogs. The use of a bipolar VAS scale in this study, instead of a unipolar scale used in other studies, could potentially be a source of discrepancy if patients perceive the two scales differently and tend to report lower liking when using a bipolar scale compared to a unipolar scale. While the FDA required the use of the bipolar scale for this pivotal study, that may have led to differences in the proportion of values exceeding the 11-point threshold.

The ability of BPN to block drug liking should also be considered for other opioids, such as methadone and fentanyl. Given the non-opioid activity of methadone and the much higher potency of fentanyl relative to hydromorphone, the results reported here should not be directly applied to the blockade of methadone and fentanyl by BPN. Recent studies have shown that BPN is effective in protecting against fentanyl-induced respiratory depression in both opioid-naïve and opioid-dependent individuals, with buprenorphine plasma concentrations ≥2 ng/mL effective against high doses of fentanyl in chronic opioid users [31, 32].

## Conclusion

The primary objective of this population PK/PD analysis was to investigate the relationship between BPN plasma concentration and drug liking E_max_ VAS score associated with intramuscular hydromorphone challenges before and after subcutaneous administration of CAM2038 Q1W 24 mg and 32 mg. PK/PD analyses for desire to use VAS score and COWS score were also performed. The PK/PD relationships were adequately described by direct I_max_ models. The drug liking E_max_ VAS score model indicated that drug liking following hydromorphone 18 mg challenge was completely blocked at low BPN plasma concentrations (0.4 ng/mL). However, other studies have reported that higher concentrations (>1.0 ng/mL) are required for complete blockade, and further analysis and replication at low concentrations are required to confirm the findings of this model. The BPN plasma concentration required for a therapeutic effect for desire to use VAS score and COWS score was in the same concentration range, but lower than that for drug liking blockade. Variability was limited among participants in individual PK/PD relationship for drug liking E_max_ VAS score with a few participants falling outside the 11-point threshold, affirming the need to individualize treatment (for example, through different formulations and dose strengths for some patients) and titrate doses for optimal treatment outcomes.

### Supplementary information

Supplementary material

## Data Availability

The authors will not make data collected for the study available to others, including individual participant data and a data dictionary defining each field in the set. The final clinical trial protocol is available as a supplement to Walsh SL et al. Effect of Buprenorphine Weekly Depot (CAM2038) and Hydromorphone Blockade in Individuals With Opioid Use Disorder: A Randomized Clinical Trial. JAMA Psychiatry. 2017; 74: 894–902.
