# Supplementary material for: Pharmacokinetic-pharmacodynamic analysis of drug liking blockade by buprenorphine subcutaneous depot (CAM2038) in participants with opioid use disorder
Source: Neuropsychopharmacology. 2024 Jan 10;49(6):1050–7. doi: 10.1038/s41386-023-01793-z (PMC11039630; doi:10.1038/s41386-023-01793-z)
Supplement: Supplementary file 1 — Supplementary material [file 41386_2023_1793_MOESM1_ESM.pdf]

SUPPLEMENTARY APPENDIX

Supplementary Methods: Drug liking maximum effect (E_{max}) visual analog scale (VAS) score

Parameter Estimation

For each parameter, interindividual variability (IIV) was initially evaluated in an exponential form ($P_i = TVP \cdot e^{\eta i}$), where TVP is the typical value of the parameter P, P_i is the individual value of the parameter and η_i is a normally distributed random variable with mean 0 and standard deviation ω. Other distributions of IIV, such as additive, logit and Box-Cox transformations were also tested.

Residual Unexplained Variability (RUV)

The residual unexplained variability (RUV; denoted below as ŷ) was initially quantified with different models, such as the additive (homoscedastic), proportional (heteroscedastic) and combined additive and proportional error model. It was established that only the additive error model allowed estimations of the baseline IIV, when compared with other RUV models. However, too many negative drug liking E_{max} VAS scores were simulated and the scores post-administration were over-predicted with this model. As such, more elaborate error models were tested and the distribution of the observations led to a logit transformation of the predictions, which was defined such that predictions could fit in the range −1 to 52.

$$\mathrm{TI\hat{y}_{ij}} = (\mathrm{\hat{y}_{ij}} + 1)/53$$

$$\mathrm{PHI\hat{y}_{ij}} = \ln(\frac{\mathrm{TI\hat{y}_{ij}}}{1 - \mathrm{TI\hat{y}_{ij}}})$$

$$\mathrm{y_{ij}} = \left(\frac{\mathrm{e}^{\left(\mathrm{PHI\hat{y}_{ij}} + \varepsilon_{\mathrm{add,ij}}\right)}}{1 + \mathrm{e}^{\left(\mathrm{PHI\hat{y}_{ij}} + \varepsilon_{\mathrm{add,ij}}\right)}}\right) * 53 - 1$$

where, for a continuous endpoint, $\hat{y}_{ij}$ is the model predictions for y_{ij} (the jth observation from the ith individual), $PHI\hat{y}_{ij}$ is the logit transformation of the model predictions, and $\varepsilon_{add,ij}$ is a normally distributed random variable with the mean 0 and standard deviation σ_{add}.

Model Evaluation

Goodness of fit plots of observed concentrations versus individual predictions (IPRED) or population predictions (PRED) were evaluated for random scatter around the line of identity, while conditional weighted residuals (CWRES) versus PRED and CWRES versus time since first CAM2038 dose were evaluated for random scatter around the horizontal line across the zero. Plots of individual weighted residuals (NIWRES) versus IPRED and NIWRES versus time since first CAM2038 dose were evaluated for random scatter.

Discrimination between models was mainly based on the inspection of graphical diagnostics and changes in the objective function values provided by non-linear mixed-effects modeling (NONMEM®). The between-model changes in objective function values were nominally χ^2 distributed and a difference of −3.84 (larger model−smaller model) corresponded to an approximate p-value of <0.05 for one degree of freedom, provided that the models were nested. For a more complicated model to be retained it had to provide a significant improvement over the contending model (p<0.05 hierarchical models) and plausible parameter estimates that were not associated with excessively high relative standard errors. It also had to demonstrate improvements in the graphical diagnostics and result in a condition number <1,000.

Hardware and Software

NONMEM® version 7.3.0 was installed on an Intel Xeon-based server running Scientific Linux 6.3. The gfortran complier (version 4.4.6) was used to perform the NONMEM® runs.

Supplementary Methods: Desire to use VAS score and Clinical Opiate Withdrawal Scale (COWS) score

Data from the phase 2 study were related with 0, 6 and 18 mg hydromorphone challenges and all data were used to inform the pharmacokinetic/pharmacodynamic (PK/PD) model development and conclusions, since included data of both desire to use VAS score and COWS score were evaluated before the hydromorphone challenge.

Desire to use VAS score was collected on pen and paper and assessed on a 100 mm unipolar (i.e., 0=no effect) scale. In this analysis, the pre-hydromorphone challenge observations of desire to use VAS score were used.

The COWS consists of 11 opioid-withdrawal signs rated by a trained observer on a scale from 0 to 4 (total scale range, 0–44, where 5–12 represents mild symptoms). In this analysis, only the total COWS score was used.

Overview of the main analysis steps

The discrimination between models was mainly based on the inspection of graphical diagnostics and on changes in the objective functional value (OFV) provided by NONMEM®. The requirements for retaining a more complex model were that it should show a significant improvement over the contending model ($p<0.05$ for nested models) as well as provide plausible parameter estimates that are not

associated with excessively high relative standard errors (RSEs). Furthermore, it should preferably have demonstrated improvements in the graphical diagnostics and not lead to a high (>1,000) condition number.

Model evaluation

Model evaluation was based on the inspection of RSEs, plausibility of the parameter estimates, graphical diagnostics, including goodness of fit plots and visual predictive checks (VPCs), as well as changes in the OFV provided by NONMEM®. For the desire to use VAS score model, stratification was applied to ensure that the final model performed adequately across important subgroups of data.

Goodness of fit plots

Plots of observations versus predictions were evaluated for random scatter around the line of identity, while CWRES plots were evaluated for random scatter around the zero line.

Visual predictive checks

Visual predictive checks (VPCs) were used to evaluate the predictive performance of the key models in the analysis.

For the desire to use VAS score model, data were simulated 1,000 times using the doses and covariate data from the participants in the analysis dataset and using the same study design. The dependent variables (DVs) of both observed and simulated data were plotted versus time and buprenorphine (BPN) plasma concentration and these profiles were graphically compared. For the observed data, the median, as well as the 5th and 95th percentiles, are presented. For the simulated data, 95%

confidence intervals (CIs) around the observed median and prediction interval are presented.

For the COWS score model, data were simulated 300 times using the doses and covariate data from the participants in the analysis dataset and using the same study design. The DVs of both observed and simulated data were plotted versus time and BPN plasma concentration and these profiles were graphically compared. For the observed data, the median, as well as the 10th and 90th percentiles, are presented. For the simulated data, 80% CIs around the observed median and prediction interval are presented.

Model application

In a separate simulation, COWS was simulated 1,000 times over the range of BPN concentration (0–10 ng/mL), which mostly covers the observed BPN plasma concentration range (0.636–12.3 ng/mL). This simulation was performed with the final model, including the IIV. The median, 50% prediction interval (PI) and 90% PI were calculated, and a predicted proportion of participants with COWS score below 5 (the lower boundary for mild symptoms) was computed.

Hardware and Software

NONMEM® version 7.5 was installed on a computer cluster running Red Had Enterprise Linux 8. NONMEM® runs were performed using the GCC compiler, version 7.5, and facilitated by Perl-speaks-NONMEM® (PsN), version 5.3.16. Data management and further processing of NONMEM® output were performed using R version 3.5.3 (2019-03-11).

Parameter estimation was performed using the first-order conditional estimation method with interaction (FOCEI) method and Monte Carlo importance sampling (IMP) method in NONMEM® for the development of desire to use VAS score and COWS score models, respectively. The standard errors of the parameter estimates were computed using the default MATRIX option and the MATRIX=R option in the NONMEM® $COV record for the desire to use VAS score and COWS score, respectively.

Model development: Desire to use VAS score

The starting model was a maximum inhibition (I_{max}) model relating daily BPN plasma concentrations with daily desire to use VAS score:

$$\mathrm{IPRED} = \mathrm{BASE} \cdot \left(1 - \frac{\mathrm{I_{max}} \cdot \mathrm{Cp}^{\gamma}}{\mathrm{IC}_{50}^{\gamma} + \mathrm{Cp}^{\gamma}}\right)$$

where IPRED is the predicted desire to use VAS score, BASE is the baseline desire to use VAS score, I_{max} is the maximum inhibition, IC_{50} is the concentration at half maximum inhibition, γ is the sigmoidicity parameter, and Cp is the time-matched BPN concentration. The pre-challenge VAS score recordings of all challenge days were used in the analysis. I_{max} was fixed to 1 (i.e. complete inhibition). The sigmoidicity parameter, γ, was fixed to 1. To keep estimates within the boundaries of 0 and 100, and still allow for predictions of exactly 0 and 100, a logit transformation of the predictions was made according to the following equations:

$$\mathrm{TI}\hat{y}_{ij} = (\hat{y}_{ij} + 1)/102$$

$$\mathrm{PHI}\hat{y}_{ij} = \ln\left(\frac{\mathrm{TI}\hat{y}_{ij}}{1 - \mathrm{TI}\hat{y}_{ij}}\right)$$

$$y_{ij} = \left(\frac{e^{\left(PHI\hat{y}_{ij} + \varepsilon_{add,ij}\right)}}{1 + e^{\left(PHI\hat{y}_{ij} + \varepsilon_{add,ij}\right)}}\right) * 102 - 1$$

where $\hat{y}_{ij}$ is the model predictions for y_{ij} (the jth observation from the ith individual), $PHI\hat{y}_{ij}$ is the logit transformation of the model predictions, and $\varepsilon_{add,ij}$ is a normally distributed random variable with the mean 0 and standard deviation σ_{add}.

In many participants a delay in the onset of the effect was observed. This was modeled by describing I_{max} as an exponential function of time, according to the following equation:

$$I_{max} = TVI_{max} \cdot (1 - e^{-kt \cdot time})$$

where TVI_{max} is the typical value of I_{max} and kt is the rate constant for onset of drug effect.

The distribution of baseline desire to use VAS score included values of 0 and 100, and therefore a logit transformation of the baseline, similar to what is described for the predictions of desire to use, was applied. The baseline distribution was also heavily skewed, which was modeled using a Box-Cox transformation of the baseline variability. This model was regarded as the final model.

The use of a mixture model, estimating the probability of being a non-responder (I_{max}=0), being a complete responder (IC_{50} fixed to a very low number), or an intermediate responder, was investigated. This model did not lead to a statistically significant improvement and was therefore not investigated further.

Model development: COWS score

The starting model was a bounded integer model for COWS (total score) using the implementation with improved numerical stability suggested by Ueckert et al [1]. An I_{max} model with a decreasing effect of BPN plasma concentration on COWS was implemented:

$$\mathrm{IPRED(Cp)} = \mathrm{BASE_{b.int}} \cdot \left(1 - \frac{\mathrm{I_{max}} \cdot \mathrm{Cp}^{\gamma}}{\mathrm{IC}_{50}^{\gamma} + \mathrm{Cp}^{\gamma}}\right)$$

where individual prediction (IPRED) is the prediction on the scale of quantile function of standard distribution, $BASE_{b.int.}$ is the baseline estimation of the COWS before the drug administration (when BPN plasma concentration [Cp] is 0) on the scale of quantile function of standard distribution, I_{max} is the maximum inhibitory effect of the BPN proportional to the $BASE_{b.int.}$, IC_{50} is the plasma concentration of BPN required to achieve half of the maximum effect and γ is the sigmoidicity parameter.

In addition, two alternative I_{max} models were tested, according to the two following equations, to allow for more flexibility in the estimation of the baseline:

$$\mathrm{IPRED(Cp)} = \mathrm{BASE_{b.int}} - \left(\frac{\mathrm{I_{max}} \cdot \mathrm{Cp}^{\gamma}}{\mathrm{IC}_{50}^{\gamma} + \mathrm{Cp}^{\gamma}}\right)$$

where I_{max} is the maximum inhibitory effect of the BPN not proportional to the $BASE_{b.int}$., and therefore represents a maximum decrease from baseline on the scale of quantile function of standard distribution, and:

$$\mathrm{IPRED(Cp)} = \mathrm{BASE_{b.int}} - \left(\frac{(\mathrm{BASE_{b.int}} - \mathrm{LBASE_{b.int}}) \cdot \mathrm{Cp}^{\gamma}}{\mathrm{IC}_{50}^{\gamma} + \mathrm{Cp}^{\gamma}}\right)$$

where $LBASE_{b.int.}$ is the parameter representing the lower baseline value of the COWS on the scale of quantile function of standard distribution, after the maximum decrease from baseline. The final model was implemented according to this equation. In addition, the sigmoidicity, γ, was fixed to 1, and the IC_{90} was estimated instead of the IC_{50}. The additive IIV on $BASE_{b.int.}$, exponential IIV on $LBASE_{b.int.}$, and the correlation between these two parameters was also estimated.

Supplementary Data: Observations versus time

Desire to use VAS score

Profiles of individual observations versus time and challenge sessions suggested a relatively rapid decrease of desire to use VAS score for most participants. The effect seemed more pronounced in the 24 mg treatment group, and similar between the hydromorphone challenge levels, which may partially be related with the fact that the pre-challenge desire to use VAS score observations were analyzed. A variability in the time of the onset of the desire to use VAS score decrease was observed, with a delay in the decrease of desire to use VAS score for some participants. Moreover, some participants stayed at the high level of desire to use VAS score during the whole observation period.

Profiles of individual observations versus BPN concentration suggested a rapid decrease of desire to use VAS score for most participants, and many achieved the minimum desire to use VAS score level already at the lowest observed BPN concentration.

COWS score

Profiles of individual observations versus time suggested a rapid decrease of the COWS score after the start of the CAM2038 24 mg and 32 mg treatment. Almost all

participants remained below the threshold of mild withdrawal (COWS score 5–12) during the whole treatment period. The effect seemed similar between the treatment groups and between the hydromorphone challenge levels, which may partially be related with the fact that COWS was evaluated on the same day before the hydromorphone challenge.

Profiles of individual COWS observations versus BPN concentration suggest a rapid decrease of COWS score for all participants, and the decrease was observed at the lowest observed BPN concentrations.

Supplementary Data: CONSORT flow diagram

The CONSORT flow diagram has been published previously in Walsh et al [2].

Table S1. Baseline characteristics

Covariate		CAM2038 24 mg (n=22)	CAM2038 32 mg (n=25)	All (N=47)
Age, years	Median (min, max)	34.5 (21.0, 53.0)	36.0 (18.0, 54.0)	36.0 (18.0, 54.0)
	Mean (SD)	36.1 (9.3)	35.6 (9.1)	35.8 (9.1)
Body weight, kg	Median (min, max)	71.0 (53.0, 110.0)	75.7 (53.9, 105.0)	73.4 (53.0, 110.0)
	Mean (SD)	75.4 (14.2)	76.4 (14.0)	75.9 (14.0)
Height, cm	Median (min, max)	174 (154, 185)	178 (158, 192)	176 (154, 192)
	Mean (SD)	173 (8.5)	177 (8.2)	175 (8.5)
BMI, kg/m^2	Median (min, max)	23.9 (20.1, 34.0)	24.5 (16.8, 34.0)	24.3 (16.8, 34.0)
	Mean (SD)	25.2 (4.3)	24.4 (4.3)	24.8 (4.2)
Sex, n (%)	Female	6 (27)	6 (24)	12 (26)
Race, n (%)	Black or African American	9 (41)	15 (60)	24 (51)
	White	12 (55)	10 (40)	22 (47)
	Other	1 (5)	0 (0)	1 (2)
Ethnicity, n (%)	Hispanic or Latino	0 (0)	1 (4)	1 (2)
	Not Hispanic or Latino	22 (100)	24 (96)	46 (98)

BMI: body mass index; n: number; SD: standard deviation.

Table S2. Analysis datasets

Randomized dose of CAM2038	Number of participants	Number of observations				
		Drug liking E_{max} VAS score			Desire to use VAS score	COWS score
		PK	PD	Total	PD	PD
CAM2038 24 mg Q1W	22	109	109	218	328	284
CAM2038 32 mg Q1W	25	122	122	244	364	314
All	47	231	231	462	692	598

COWS: Clinical Opiate Withdrawal Scale; E_{max}: maximum effect; PD: pharmacodynamic; PK: pharmacokinetic; Q1W: once weekly; VAS: visual analog scale.

Table S3. Parameter estimates of the final desire to use VAS score PK/PD model

	Unit	Value	RSE (%)	Shrinkage (%)
Baseline	VAS units	92.5	0.296	
IC_{50}	ng/mL	0.0129	12.3	
I_{max}		1.00	FIXED	
Onset half-life	day	0.330	18.7	
Baseline Box-Cox shape parameter		−12.9	4.91	
IIV Baseline	CV	0.167	14.1	5.76
IIV IC_{50}	CV	9.29	21.7	42.2
IIV onset half-life	CV	1.91	17.6	33.9
RUV	CV	0.783	8.45	5.68

CV: coefficient of variation; IC_{50}: concentration at 50% of maximum inhibition; IIV: interindividual variability; I_{max}: maximum inhibition; PD: pharmacodynamic; PK: pharmacokinetic; RSE: residual standard error; RUV: residual unexplained variability; VAS: visual analog scale.

Table S4. Parameter estimates of the final COWS score PK/PD model

	Unit	Value	RSE (%)
$BASE_{b.int}$		−0.887	8.67
IC_{90}	ng/mL	0.109	43.1
$LBASE_{b.int}$		−2.11	4.58
$SD_{b.int}$		0.265	5.66
IIV $BASE_{b.int}$	CV	0.454	15.2
IIV $LBASE_{b.int}$	CV	0.0948	14.3
IIV $BASE_{b.int}$ and IIV $LBASE_{b.int}$ correlation	CV	0.0203	478
IC_{50}	ng/mL	0.012	N/A

$BASE_{b.int}$: baseline estimation of the COWS score before the drug administration (when BPN plasma concentration (Cp) is 0) on the scale of quantile function of standard distribution; BPN: buprenorphine; COWS: Clinical Opiate Withdrawal Scale; Cp: time-matched BPN concentration; CV: coefficient of variation; IC_{50}: concentration at 50% of maximum inhibition; IC_{90}: concentration at 90% of maximum inhibition; $LBASE_{b.int}$: parameter representing the lower baseline value of the COWS on the scale of quantile function of standard distribution, after the maximum decrease from baseline; IIV: interindividual variability; PD: pharmacodynamic; PK: pharmacokinetic; RSE: relative standard error; $SD_{b.int}$: scaling parameter in the bounded integer model.

Figure S1. Clinical study design

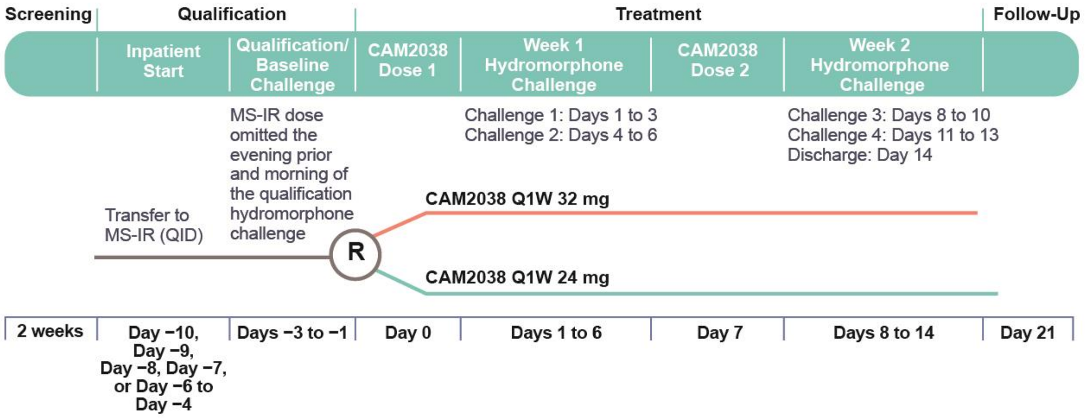

MS-IR: morphine sulphate immediate release; QID: four times daily; Q1W: once weekly; R: randomization.

Figure S2. Observed BPN plasma concentration versus time

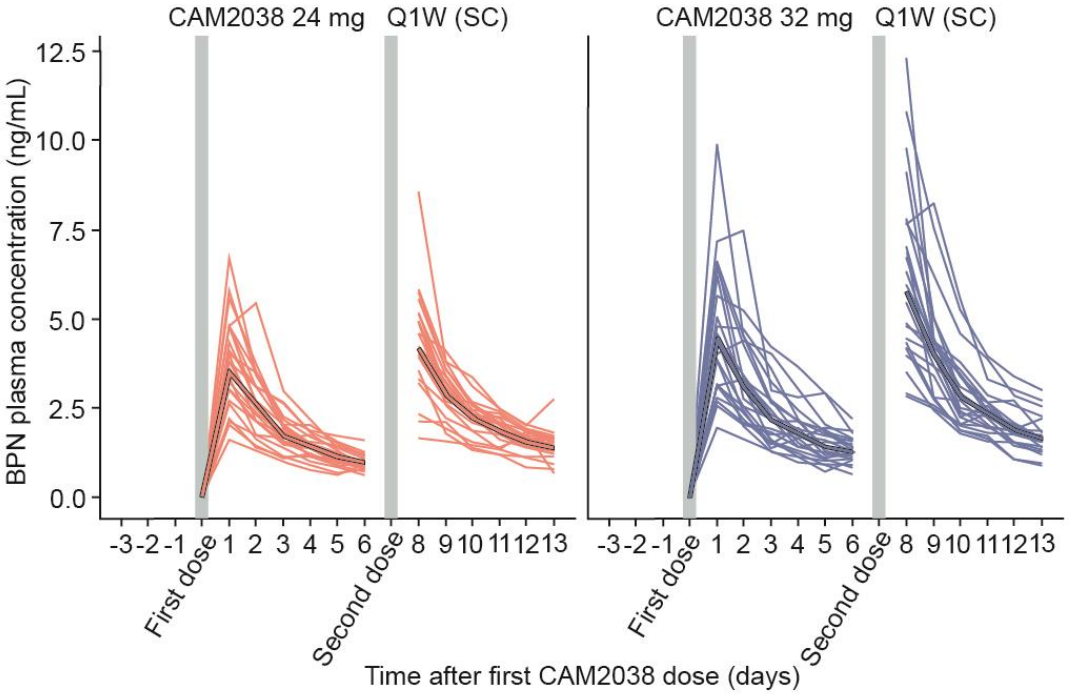

BPN plasma concentrations are stratified by CAM2038 dose at randomization. Each line represents the data for one participant and is colored by the dose groups. The thick line represents the geometric mean for the panel. The vertical grey lines indicate the CAM2038 administrations. BPN: buprenorphine; Q1W: once weekly; SC: subcutaneous.

Figure S3. Period-corrected drug liking E_{max} VAS score superimposed with BPN plasma concentrations versus hydromorphone challenge sessions.

A)

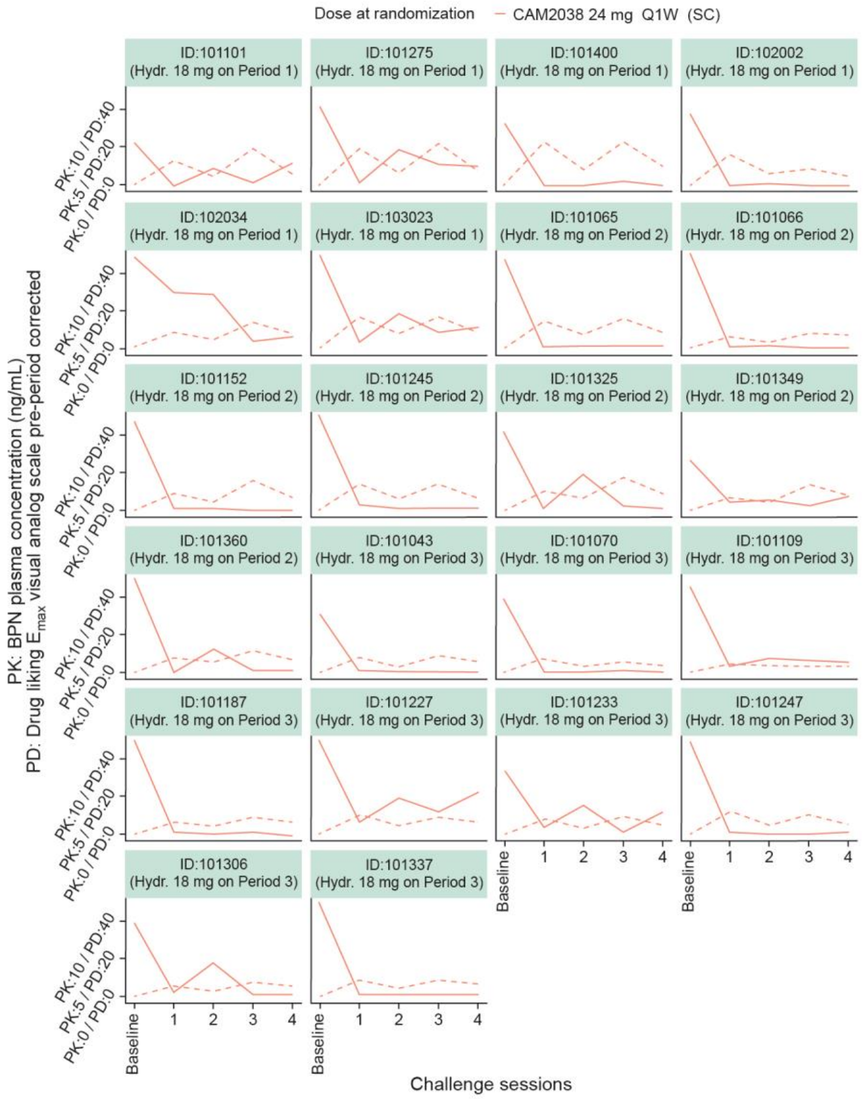

B)

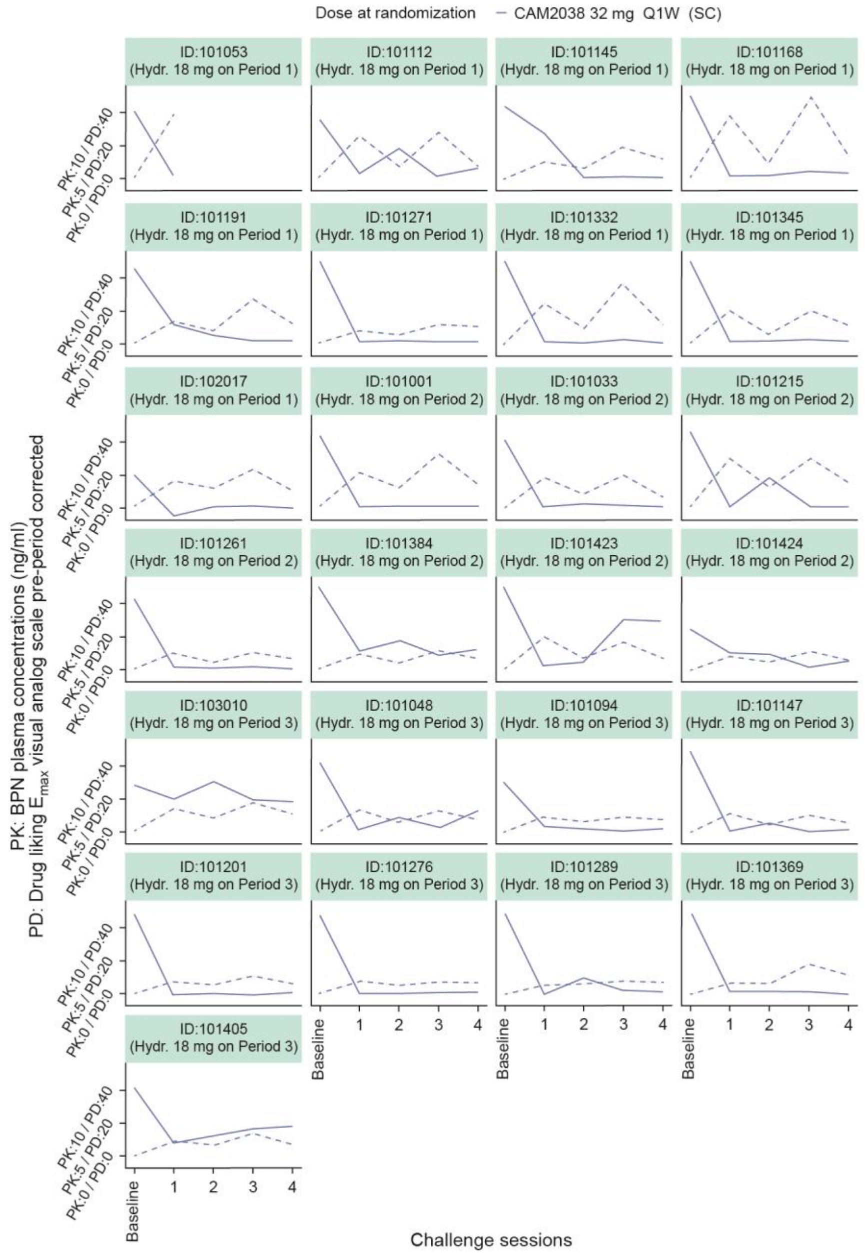

A) Observed period-corrected drug liking E_{max} VAS score superimposed with BPN plasma concentrations versus challenge sessions for 24 mg CAM2038 at randomization and the 18 mg hydromorphone challenge. Each panel represents the data for one participant and is colored by the CAM2038 dose at randomization. Observed period-corrected drug liking E_{max} VAS values are displayed with full lines, BPN plasma concentrations are displayed with dotted lines. B) Observed period-corrected drug liking E_{max} VAS score superimposed with BPN plasma concentrations versus challenge sessions for 32 mg CAM2038 at randomization and the 18 mg hydromorphone challenge. Each panel represents the data for one participant and is colored by the CAM2038 dose at randomization. Observed period-corrected drug liking

E_{max} VAS values are displayed with full lines, BPN plasma concentrations are displayed with dotted lines. BPN: buprenorphine; E_{max}: maximum effect; PD: pharmacodynamic; PK: pharmacokinetic; Q1W: once weekly; SC: subcutaneous.

Figure S4. Goodness of fit plots for the final drug liking E_{max} VAS score PK/PD model

A)

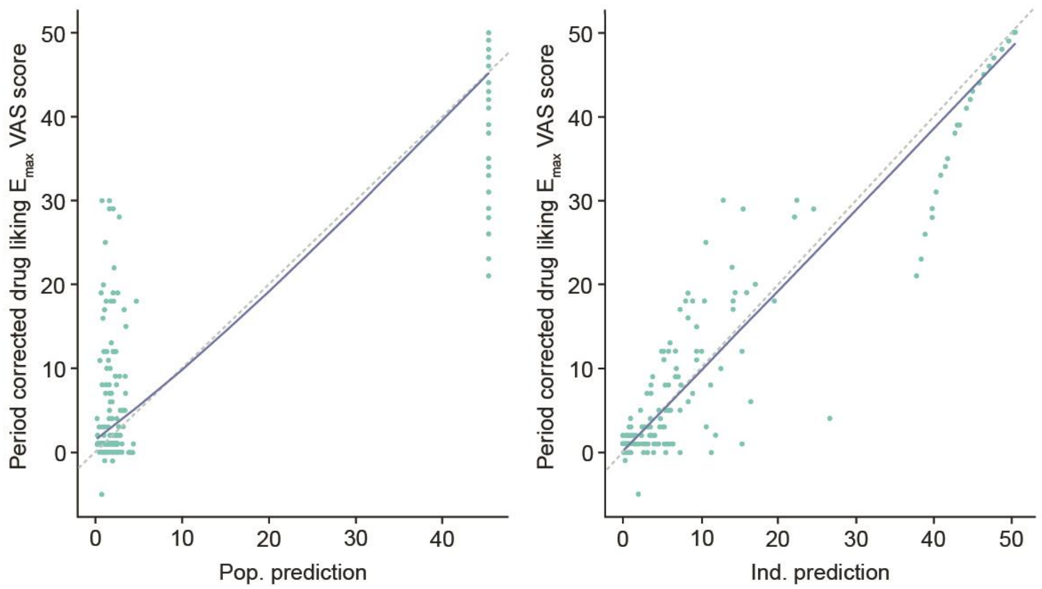

B)

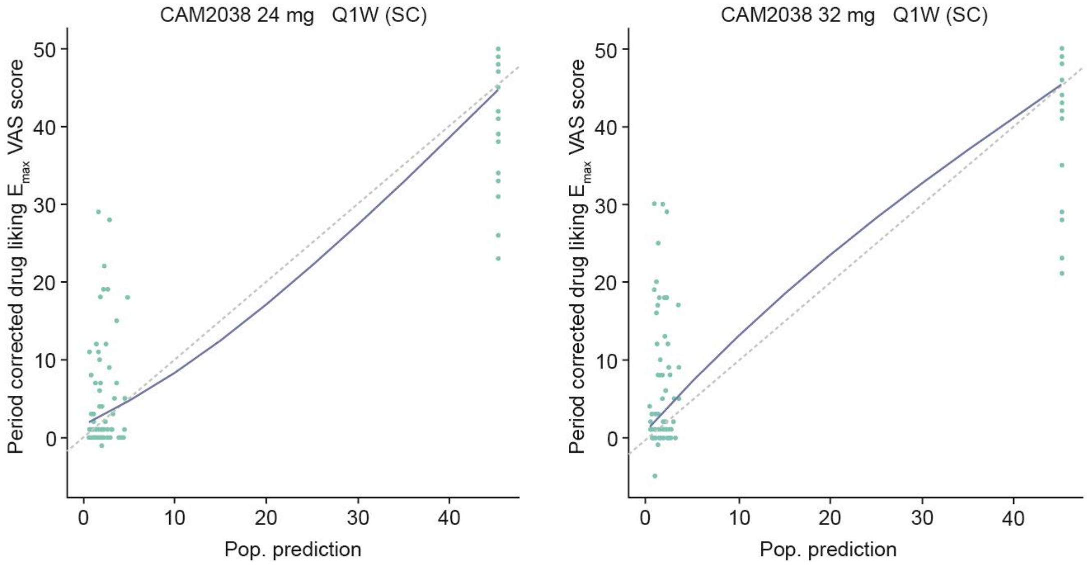

C)

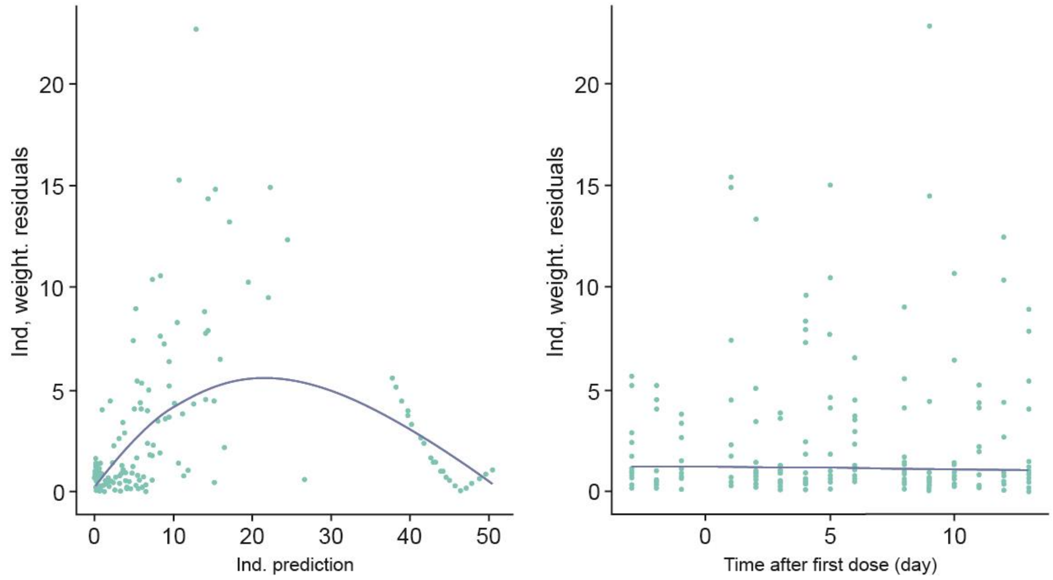

D)

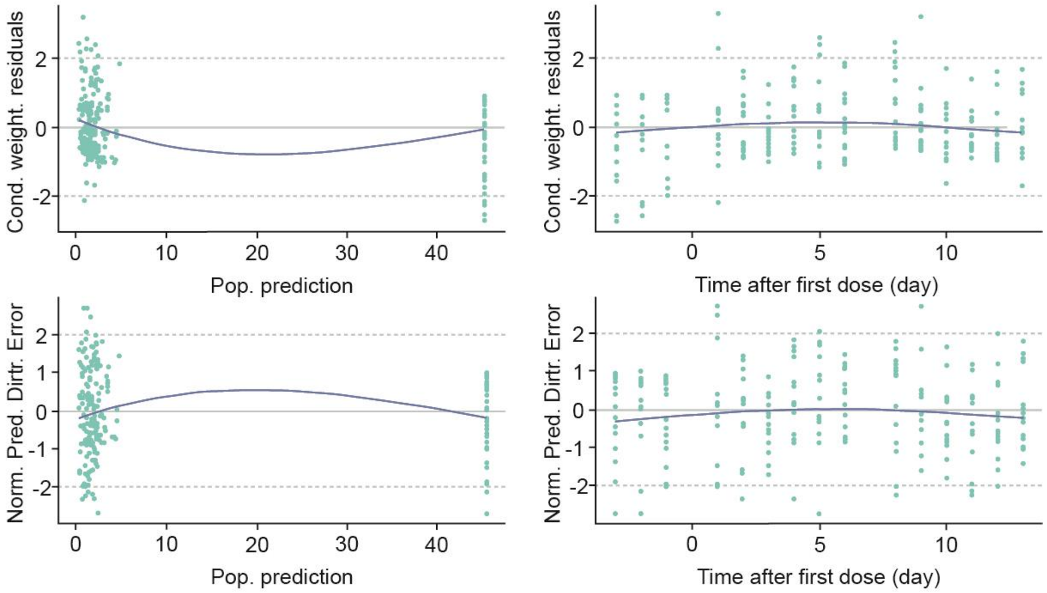

A) DV versus population and individual predictions of the final model. Individual data points are indicated by dots. The diagonal black dotted line is the line of identity and the solid blue line is a smooth. B) DV versus population predictions of the final model, stratified by dose. Individual data points are indicated by dots. The diagonal black dotted line is the line of identity and the solid blue line is a smooth. C) NIWRES versus individual predictions and versus time for the final model. Individual data points are indicated by dots. The solid blue line is a smooth. D) CWRES versus population predictions and versus time for the final model.

Individual data points are indicated by dots. The solid blue line is a smooth. CWRES: conditional weighted residuals; DV: dependent variable; Emax: maximum effect; NIWRES: individual weighted residuals; Q1W: once weekly; SC: subcutaneous; VAS: visual analog scale.

Figure S5. Visual predictive check of desire to use VAS score

A) Visual predictive check of prediction-corrected desire to use VAS score versus time

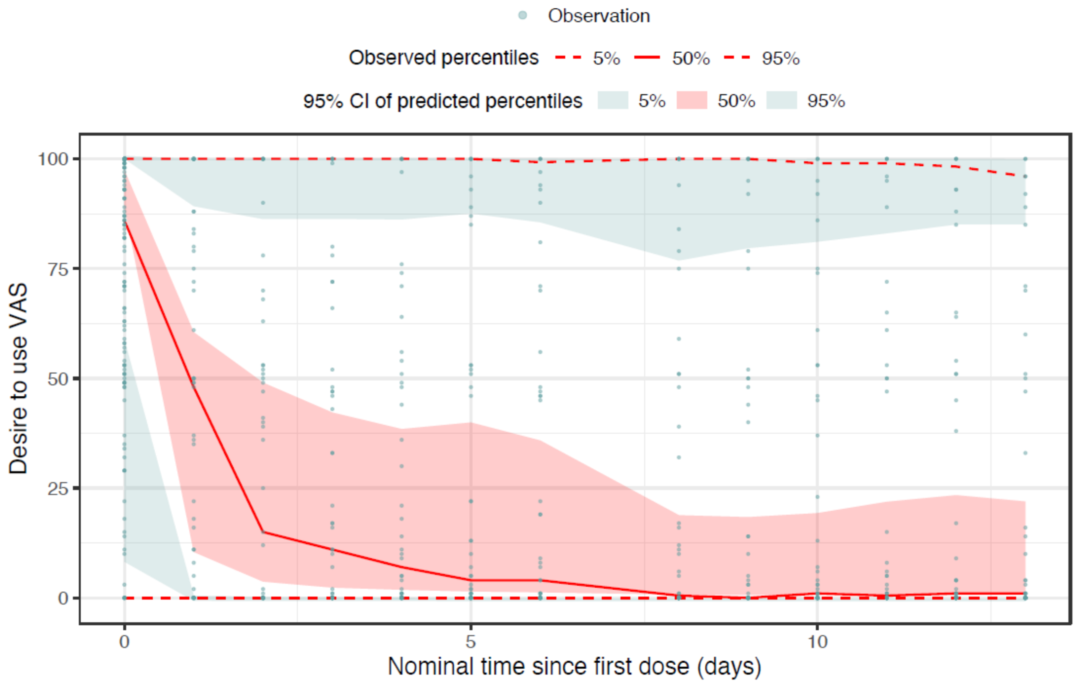

B) Visual predictive check of prediction-corrected desire to use VAS score versus BPN plasma concentration

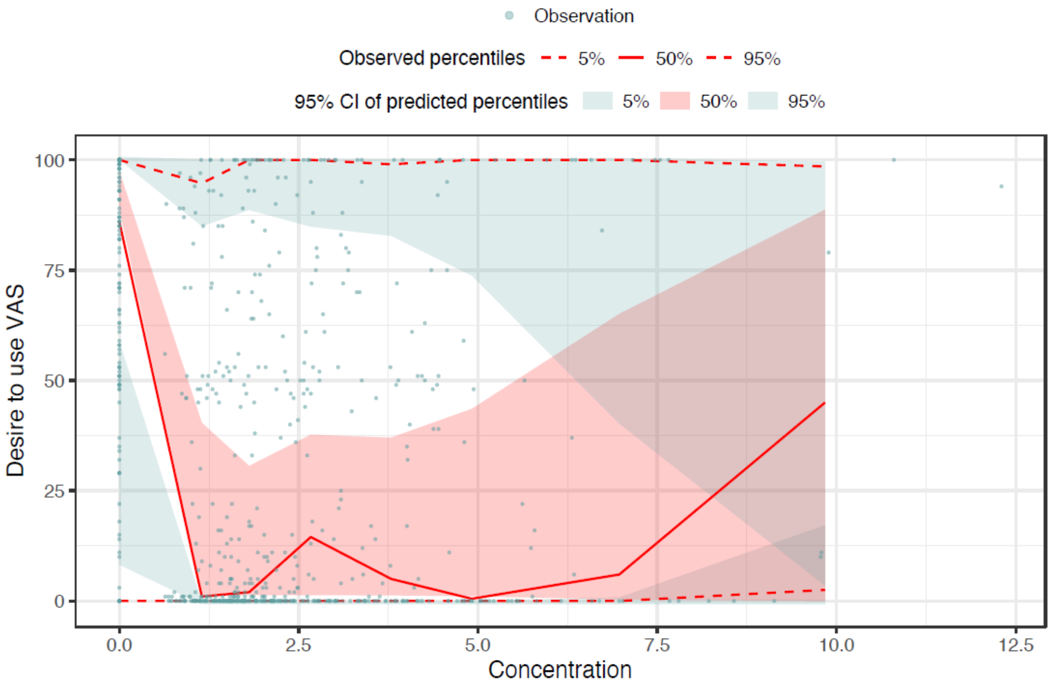

Desire to use score was prediction corrected. The solid and dashed red lines represent the median, 5th and 95th percentiles of the observations; the shaded red and blue areas represent the 95% confidence

interval of the median, 5th and 95th percentiles predicted by the model. BPN: buprenorphine; CI: confidence interval; VAS: visual analog scale.

Figure S6. Visual predictive check of COWS score

A) Visual predictive check of prediction-corrected COWS score versus time

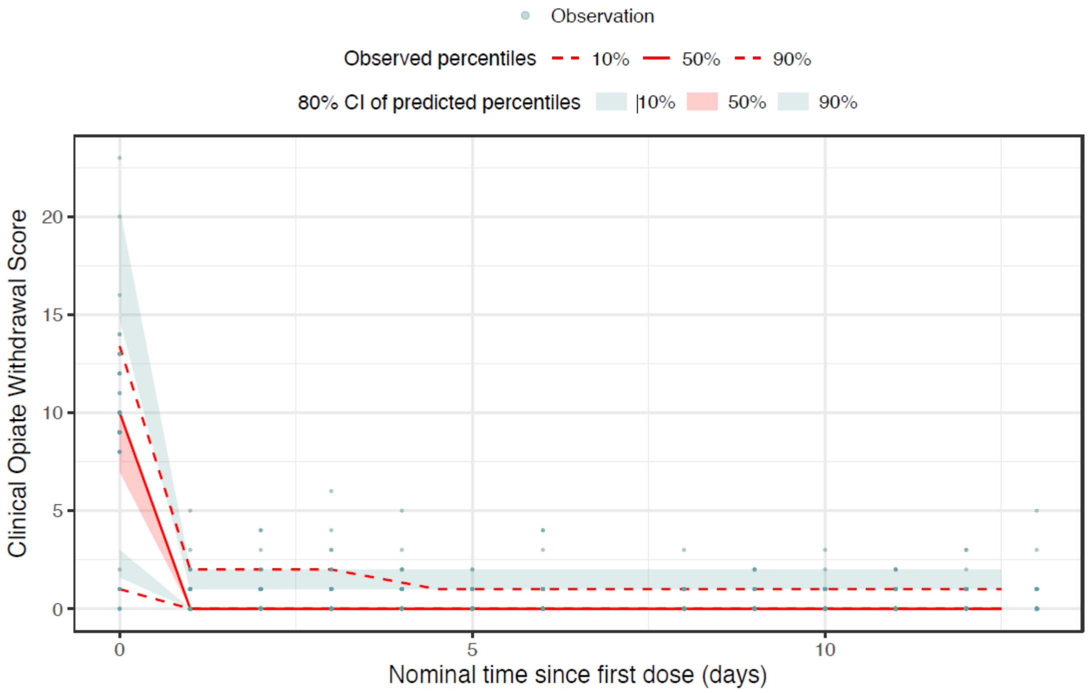

B) Visual predictive check of prediction-corrected COWS score versus BPN plasma concentration

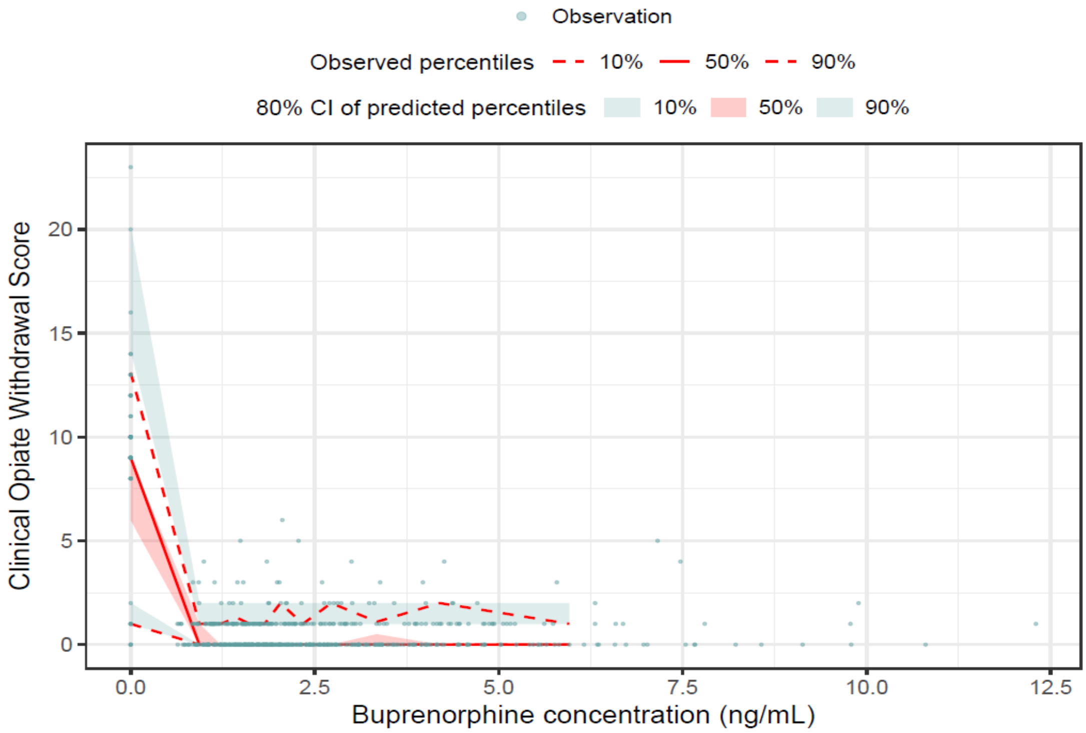

COWS score was prediction corrected. The solid and dashed red lines represent the median, 10th and 90th percentiles of the observations; the shaded red and blue areas represent the 80% confidence

interval of the median, 10th and 90th percentiles predicted by the model. BPN: buprenorphine; CI: confidence interval; COWS: Clinical Opiate Withdrawal Scale.

Figure S7. Application of the final COWS score model

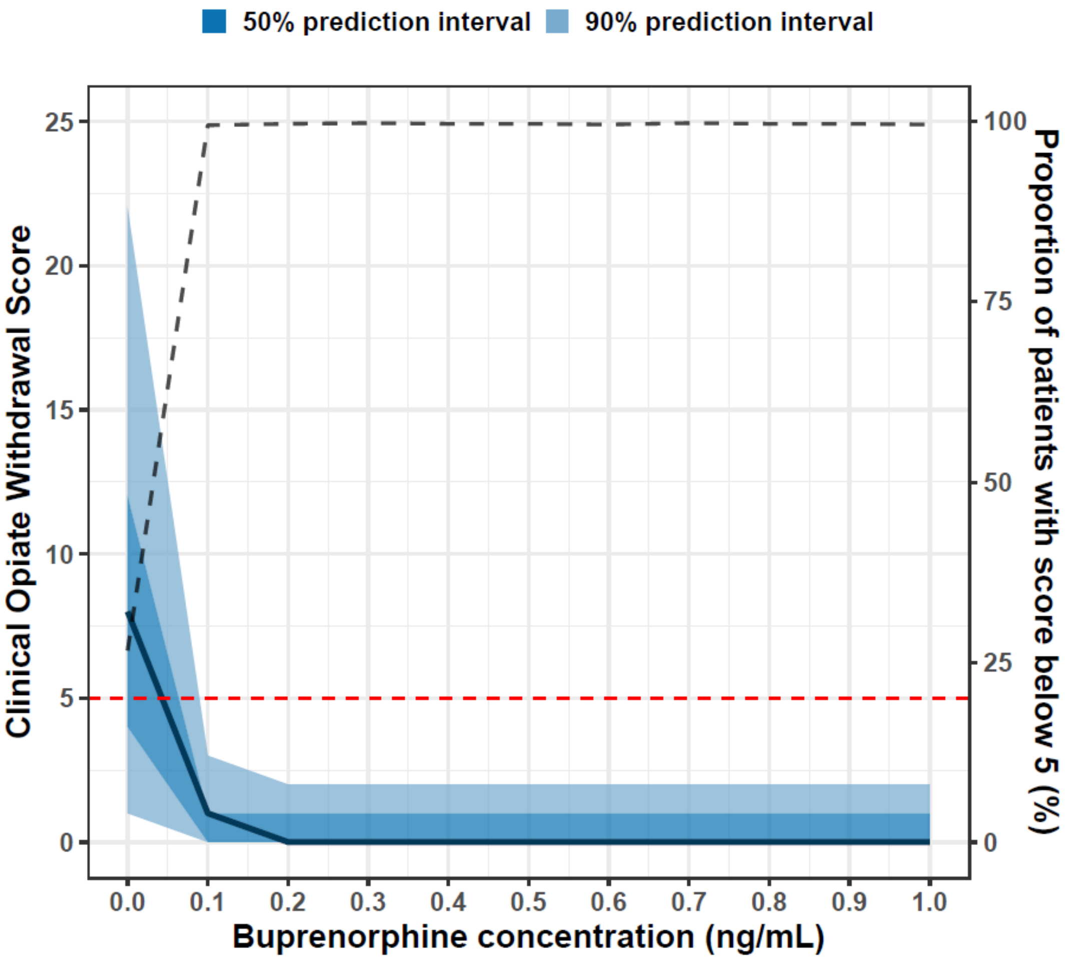

The final COWS model was used to simulate COWS score versus BPN plasma concentration, and the proportion of participants with COWS score below 5, based on 1,000 simulated participants. The solid black line represents the model predicted COWS score; the dashed black line represents the proportion of participants with COWS score below 5; the dashed red line represents the lower threshold of mild COWS symptoms; the shaded darker and lighter blue areas represent the 50% and 90% prediction intervals, respectively. BPN: buprenorphine; COWS: Clinical Opiate Withdrawal Scale.

CONSORT Flowchart

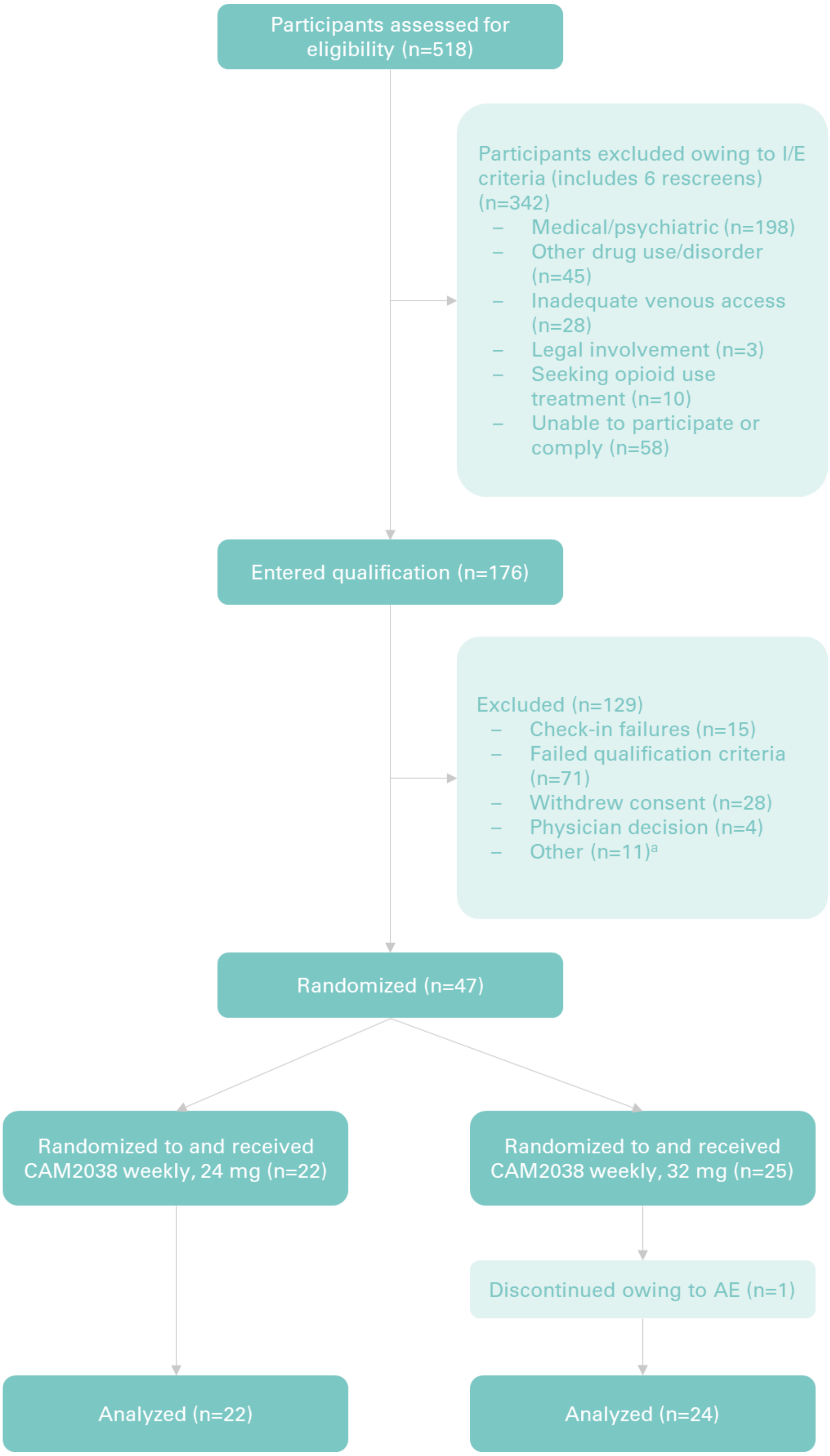

There were 114 failures at the qualification phase. [a] Other includes those leaving against medical advice or clinical laboratory and vital sign abnormalities. AE: adverse event; I/E: inclusion/exclusion. Figure adapted from Walsh et al. 2017.

REFERENCES

1 Ueckert S, Karlsson MO. Improved numerical stability for the bounded integer model. Journal of Pharmacokinetics and Pharmacodynamics. 2021 Apr;48:241-51.

2 Walsh SL, Comer SD, Lofwall MR, Vince B, Levy-Cooperman N, Kelsh D, et al. Effect of Buprenorphine Weekly Depot (CAM2038) and Hydromorphone Blockade in Individuals With Opioid Use Disorder: A Randomized Clinical Trial. JAMA Psychiatry. 2017;74(9):894–902.
